# Supplementary material for: Glutamatergic supramammillary nucleus neurons respond to threatening stressors and promote active coping
Source: eLife. 2024 Jun 3;12:RP90972. doi: 10.7554/eLife.90972 (PMC11147510; doi:10.7554/eLife.90972)
Supplement: Supplementary file 1. — Statistical analyses conducted for all figures including the type of analysis(es), p values, statistic (i.e. F, t statistic), and/or multiple comparisons. [file elife-90972-supp2.docx]

| **Figure** | **Panel** | **Test** | **Statistic** | **P value** | **Bonferroni’s Multiple comparisons** | **Multiple comparisons statistic** | **Adjusted P value** |
| --- | --- | --- | --- | --- | --- | --- | --- |
| **3** | G | T-Confidence Interval | 95% CI  -30 to 0s  [-0.73511383 -0.724353688]  1 to 30s  [1.903893784 1.965866945]  Difference  [2.630811087 2.694498841]  99% CI  -30 to 0s  [-0.737419984 -0.718129215]  1 to 30s  [1.894157095 1.975603635]  Difference  [2.620805016 2.704550491]  99.9% CI  -30 to 0s  [-0.74009622 -0.715452978]  1 to 30s  [1.882857892 1.986902837]  Difference  [2.609193202 2.716116722] | Sig at 95%  Sig at 99%  Sig at 99.9% | N/A | N/A | N/A |
|  | I | T-Confidence Interval | 95% CI  -3 to -1s  [0.213736241 0.298523885]  1 to 3s  [5.190328104 6.484427377]  Difference  [4.932810735 6.22968462]  99% CI  -3 to -1s  [0.200415137 0.311844989]  1 to 3s  [4.987010372 6.687745109]  Difference  [4.72905708 6.433438275]  99.9% CI  -3 to -1s  [0.184956303 0.327303822]  1 to 3s  [4.751064858 6.923690623]  Difference  [4.492605688 6.669889668] | Sig at 95%  Sig at 99%  Sig at 99.9% | N/A | N/A | N/A |
|  | J | T-Confidence Interval | 95% CI  -3 to -1s  [1.116192035 1.439691613]  1 to 3s  [0.049420441 0.369842849]  Difference  [-1.295973579 -0.840646778]  99% CI  -3 to -1s  [1.065366568 1.49051708]  1 to 3s  [-0.000921568 0.420184858]  Difference  [-1.367510605 -0.769109753]  99.9% CI  -3 to -1s  [1.006384792 1.549498856]  1 to 3s  [-0.059342302 0.478605592]  Difference  [-1.450527664 -0.686092694] | Sig at 95%  Sig at 99%  Sig at 99.9% | N/A | N/A | N/A |

|  | K | T-Confidence Interval | 95% CI  -3 to -1s  [-0.21380383 0.000116753]  1 to 3s  [-0.381118104 -0.039309855]  Difference  [-0.304985727 0.098244846]  99% CI  -3 to -1s  [-0.24741319 0.033726113]  1 to 3s  [-0.434820072 0.014392113]  Difference  [-0.368337842 0.16159696]  99.9% CI  -3 to -1s  [-0.286416073 0.072728995]  1 to 3s  [-0.497139961 0.076712002]  Difference  [-0.441856502 0.235115621] | ns  ns  ns | N/A | N/A | N/A |
| --- | --- | --- | --- | --- | --- | --- | --- |
| **4** | C | Two-Way RM ANOVA | Stimulation Period x Genotype F (2, 58) = 12.82  Stimulation Period  F (1.012, 29.36) = 12.27  Genotype  F (1, 29) = 11.78 | p<0.001****  p=0.0014**  p=0.0018** | Pre-stimulation  Stimulation  Post-stimulation | t=1  t=3.429  t=1 | p=0.9995  p=0.0122*  p=0.9995 |
|  | D | Two-Way RM ANOVA | Stimulation Period x Genotype F (2, 58) = 21.04  Stimulation Period  F (1.794, 52.02) = 18.34  Genotype  F (1, 29) = 0.6615 | p<0.001****  p<0.001****  p=0.4227 | Pre-stimulation  Stimulation  Post-stimulation | t=1.02  t=3.74  t=3.281 | p=0.9495  p=0.0039**  p=0.0139* |
|  | E | Two-Way RM ANOVA | Stimulation Period x Genotype F (2, 58) = 14.84  Stimulation Period  F (1.667, 48.34) = 6.658  Genotype  F (1, 29) = 4.707 | p<0.0001****  p=0.0045**  p=0.0384* | Pre-stimulation  Stimulation  Post-stimulation | t=0.7131  t=3.267  t=3.81 | p>0.9999  p=0.0156*  p=0.0052** |
| **5** | C | Two-Way ANOVA | Frequency x Genotype  F (4, 188) = 36.76  Frequency  F (4, 188) = 34.61  Genotype  F (1, 188) = 352.1 | p<0.0001****  p<0.0001****  p<0.0001**** | Cre+ vs Cre-  1 Hz  5 Hz  10 Hz  20 Hz | t=4.477  t=10.09  t=12.36  t=15.09 | p<0.0001****  p<0.0001****  p<0.0001****  p<0.0001**** |
|  | D | Two-Way Mixed Model ANOVA | Time x Genotype  F (9.116, 218.1) = 1.418  Time  F (1, 24) = 0.02824  Genotype  F (29, 694) = 1.038 | p=0.1807  p=0.868  p=0.4125 | N/A | N/A | N/A |
|  | E | Two-Way Mixed Model ANOVA | Time x Genotype  F (29, 694) = 1.035  Time  F (7.228, 173.0) = 1.030  Genotype  F (1, 25) = 90.74 | p=0.4159  p=0.4124  p<0.0001**** | N/A | N/A | N/A |
|  | F | Two-Way ANOVA | Frequency x Genotype  F (4, 115) = 12.37  Frequency  F (4, 115) = 12.38  Genotype  F (1, 115) = 187.5 | p<0.0001****  p<0.0001****  p<0.0001**** | Cre+ vs Cre-  1 Hz  5 Hz  10 Hz  20 Hz | t=4.117  t=7.622  t=8.789  t=9.156 | p=0.0004***  p<0.0001****  p<0.0001****  p<0.0001**** |
|  | H | Two-Tailed T-Test  Two-Tailed T-Test | Center Cre+ vs Cre-  t=1.407, df=36  Perimeter  t=1.356, df=36 | p=0.1681  p=0.1837 | N/A | N/A | N/A |
|  | I | Two-Tailed T-Test | Total Distance  t=4.857, df=33 | p<0.0001**** | N/A | N/A | N/A |

|  | K | Two-Tailed T-Test | Time in dark eYFP vs ChR2  t=0.4208, df=15 | p=0.6799 | N/A | N/A | N/A |
| --- | --- | --- | --- | --- | --- | --- | --- |
|  | L | Two-Tailed T-Test | Distance eYFP vs ChR2 t=3.516, df=15 | p=0.0031** | N/A | N/A | N/A |
| **6** | C | Two-Way RM ANOVA | Day x Genotype  F (3, 60) = 18.15  Day  F (1.945, 38.89) = 8.547  Genotype  F (1, 20) = 255.7 | p<0.0001****  p=0.0009***  p<0.0001**** | Cre+ vs Cre-  Day1  Day 2  Day 3  Day 4 | t=6.368  t=14.31  t=20.64  t=10.93 | p=0.0003***  p<0.0001****  p<0.0001****  p<0.0001**** |
|  | D | Two-Way RM ANOVA | Day x Genotype  F (3, 60) = 0.9819  Day  F (2.047, 40.94) = 1.251  Genotype  F (1, 20) = 41.10 | p=0.4074  p=0.2975  p<0.0001**** | Cre+ vs Cre-  Day1  Day 2  Day 3  Day 4 | t=4.756  t=5.669  t=4.839  t=3.798 | P<0.0001****  p<0.0001****  p<0.0001****  p<0.0011** |
|  | E | Mixed-Model RM ANOVA | Day x Genotype  F (3, 72) = 3.449  Day  F (1.871, 44.91) = 0.6416  Genotype  F (1, 72) = 51.92 | p=0.021*  p=0.5212  p<0.0001**** | Cre+ vs Cre-  Day1  Day 2  Day 3  Day 4  Cre+ Day Comparisons  Day1 vs. Day 2  Day1 vs. Day 3  Day1 vs. Day 4 | t=2.133  t=3.246  t=4.432  t=2.903  t=3.099  t=3.387  t=3.241 | p=0.0455*  p=0.0087**  p=0.003**  p=0.0336*  p=0.0338*  p=0.0208*  p=0.0265* |
|  | F | Two-Tailed T-Test  Two-Tailed T-Test  Two-Tailed T-Test | Active nose poke  t=12.54, df=20  Inactive nose poke  t=3.709, df=20  Rewards  t=18.78, df=20 | p<0.001****  p=0.0014**  p<0.001**** | N/A | N/A | N/A |
| **7** | B | T-Confidence Interval | 95% CI  -60 to 0s  [0.959541188 1.952620738]  0 to 60s  [-0.355286068 0.726943754]  Difference  [-2.004661471 -0.535842769]  99% CI  -60 to 0s  [0.803517083 2.108644843]  0 to 60s  [-0.525316696 -0.896974382]  Difference  [-2.235429614 -0.305074626]  99.9% CI  -60 to 0s  [0.622454726 2.2897072]  0 to 60s  [-0.722633299 1.094290984]  Difference  [-2.503230695 -0.037273545] | Sig at 95%  ns  ns | N/A | N/A | N/A |
|  | D | T-Confidence Interval | 95% CI  -10 to 0s  [-0.506739492 -0.126437473]  0 to 10s  [0.197230326 0.726601455]  Difference  [0.452596634 1.104412112]  99% CI  -10 to 0s  [-0.566489269 -0.066687695]  0 to 10s  [0.114060094 0.809771688]  Difference  [0.350189 1.206819746]  99.9% CI  -10 to 0s  [-0.635827501 0.002650537]  0 to 10s  [0.017542967 0.906288814]  Difference  [0.231347315 1.325661431] | Sig at 95%  Sig at 99%  Sig at 99.9% | N/A | N/A | N/A |
|  | E | T-Confidence Interval | 95% CI  -10 to 0s  [-0.265038212 0.2702523]  0 to 10s  [-0.237081362 0.254877431]  Difference  [-0.357219458 0.36980144]  99% CI  -10 to 0s  [-0.349138447 0.354352535]  0 to 10s  [-0.31437369 0.332169759  Difference  [-0.47144272 0.484024702]  99.9% CI  -10 to 0s  [-0.44673482 0.451948908]  0 to 10s  [-0.404069645 0.421865715]  Difference  [-0.603996167 0.616578149] | ns  ns  ns | N/A | N/A | N/A |
|  | H | Two-Way RM ANOVA | Stimulation Period x Genotype  F (2, 36) = 8.208  Stimulation Period  F (1.701, 30.62) = 12.93  Genotype  F (1, 18) = 10.11 | 0.0012**  0.0002***  0.0052** | Pre-stimulation  Stimulation  Post-stimulation | t=0.7599  t=5.2  t=2.677 | p>0.9999  p=0.0002****  p=0.0465* |
|  | I | Two-Way RM ANOVA | Stimulation Period x Genotype  F (2, 40) = 14.09  Stimulation Period  F (1.995, 39.90) = 10.01  Genotype  F (1, 20) = 1.898 | p<0.0001****  p=0.0003***  p=0.1835 | Pre-stimulation  Stimulation  Post-stimulation | t=1.923  t=0.7005  t=4.238 | p=0.2183  p>0.9999  p=0.0012** |
| **8** | C | Two-Tailed T-Test | Fed vs Food Deprived  t=9.666, df=15 | p<0.0001**** | N/A | N/A | N/A |
|  | D | Two-Tailed T-Test | Fed vs Food Deprived  t=6.807, df=15 | p<0.0001**** | N/A | N/A | N/A |
|  | E | T-Confidence Interval | Isosbestic  95% CI  -150 to -10s  [-0.127314348 0.690501429]  10 to 150s  [0.709907655 1.43150582]  Difference  [0.243786104 1.334440291]  99% CI  -150 to -10s  [-0.255802519 0.818989599]  10 to 150s  [0.596536367 1.544877109]  Difference  [0.072431912 1.505794483]  99.9% CI  -150 to -10s  [-0.404910063 0.968097143]  10 to 150s  [0.464971614 1.676441861]  Difference  [-0.126420656 1.704647051]  Ca^2+^ -Dependent  95% CI  -150 to -10s  [0.024359471 0.477339678]  10 to 150s  [0.564052895 1.293569678]  Difference  [0.248605856 1.107317568]  99% CI  -150 to -10s  [-0.046808878 0.548508026]  10 to 150s  [0.449437502 1.408185071]  Difference  [0.113692468 1.242230956]  99.9% CI  -150 to -10s  [-0.129398096 0.631097245]  10 to 150s  [0.316428994 1.541193579]  Difference  [-0.042871391 1.398794816] | Sig at 95%  ns  ns  Sig at 95%  ns  ns | N/A | N/A | N/A |
|  | F | T-Confidence Interval | Isosbestic  95% CI  -150 to -10s  [0.049394291 0.689096421]  10 to 150s  [0.670463682 1.423655887]  Difference  [0.183719975 1.171908881]  99% CI  -150 to -10s  [-0.051110197 0.78960091]  10 to 150s  [0.55212861 1.541990959]  Difference  [0.028464246 1.32716461]  99.9% CI  -150 to -10s  [-0.167743327 0.906234039]  10 to 150s  [0.414803502 1.679316067]  Difference  [-0.151706429 1.507335286]  Ca^2+^ -Dependent  95% CI  -150 to -10s  [-0.875926952 0.142125648]  10 to 150s  [1.203199181 1.724507405]  Difference  [1.258872541 2.402635349]  99% CI  -150 to -10s  [-1.035874608 0.302073304]  10 to 150s  [1.121295723 1.806410863]  Difference  [1.07917438 2.582333509]  99.9% CI  -150 to -10s  [-1.221490155 0.487688851]  10 to 150s  [1.026248658 1.901457927]  Difference  [0.87063883 2.79086906] | ns  ns  ns  Sig at 95%  Sig at 99%  Sig at 99.9% | N/A | N/A | N/A |
|  | H | Two-Way RM ANOVA | Stimulation Period x Genotype  F (2, 62) = 8.738  Stimulation Period  F (1.911, 59.23) = 13.36  Genotype  F (1, 31) = 18.60 | p=0.0005***  p<0.0001****  p=0.0002*** | Cre+ vs Cre-  Pre-stimulation  Stimulation  Post-stimulation  Cre+  Pre stim vs. Stim  Pre stim vs. Post stim  Stim vs. Post stim | t=1.333  t=4.733  t=5.233  t=3.516  t=3.396  t=3.463 | P=0.4729  p=0.0006***  p=0.0002***  p=0.0186*  p=0.0074**  p=0.0207* |
|  | I | Two-Tailed T-Test | Cre+ vs Cre-  t=3.621, df=31 | p=0.001** | N/A | N/A | N/A |
| **Supp Fig 4** | C | Mann-Whitney | Control vs FST | p=0.0286* | N/A | N/A | N/A |
|  | G | Mann-Whitney | Control vs FST | p=0.0159* | N/A | N/A | N/A |
| **Supp Fig 6** | D | Two-Way RM ANOVA | Stimulation Period x Genotype  F (1, 29) = 0.002860  Stimulation Period  F (1, 29) = 0.04236  Genotype  F (1, 29) = 1.790 | p=0.9577  p=0.8384  p=0.1914 | Cre+  Baseline vs 10Hz  Cre+ vs Cre-  Baseline  10 Hz | t=0.1864  t=0.8956  t=0.9722 | p>0.9999  p=0.7483  p=0.67 |
|  | F | Two-Tailed T-Test  Two-Tailed T-Test | Center  t=1.175, df=19  Perimeter  t=1.094, df=19 | p=0.254425  p=0.287443 | N/A | N/A | N/A |
|  | G | Two-Tailed T-Test | Distance  t=2.278, df=19 | p=0.0345* | N/A | N/A | N/A |
| **Supp Fig 7** | A | Two-Way RM ANOVA | Day x Activity State  F (3, 60) = 0.99  Day  F (2.0, 41) = 1.2  Activity state  F (1, 20) = 40 | p=0.41  p=0.3  p=0.001** | N/A | N/A | N/A |
|  | B | One-Way RM ANOVA | Day  F (2.537, 25.37) = 3.042 | p=0.0546 | N/A | N/A | N/A |
